# Theorizing the Role of Dopaminergic Polymorphic Risk Alleles with Intermittent Explosive Disorder (IED), Violent/Aggressive Behavior and Addiction: Justification of Genetic Addiction Risk Severity (GARS) Testing

**DOI:** 10.3390/jpm12121946

**Published:** 2022-11-23

**Authors:** Edward Justin Modestino, Kenneth Blum, Catherine A. Dennen, B. William Downs, Debasis Bagchi, Luis Llanos-Gomez, Igor Elman, David Baron, Panayotis K. Thanos, Rajendra D. Badgaiyan, Eric R. Braverman, Ashim Gupta, Mark S. Gold, Abdalla Bowirrat

**Affiliations:** 1 The Kenneth Blum Behavioral & Neurogenetic Institute, Austin, TX 78701, USA; 2 Department of Psychology, Curry College, Milton, MA 02360, USA; 3 Division of Addiction Research & Education, Center for Psychiatry, Medicine & Primary Care, Western University Health Sciences, Pomona, CA 91766, USA; 4 Department of Precision Behavioral Management, Geneus Health, San Antonio, TX 78283, USA; 5 Institute of Psychology, ELTE Eötvös Loránd University, H-1053 Budapest, Hungary; 6 Department of Psychiatry, University of Vermont, Burlington, VT 05401, USA; 7 Centre for Genomics and Applied Gene Technology, Institute of Integrative Omics and Applied Biotechnology, Nonakuri, Purba Medinipur 721172, West Bengal, India; 8 Department of Psychiatry, Wright State University Boonshoft School of Medicine and Dayton VA Medical Centre, Dayton, OH 45324, USA; 9 Department of Psychiatry, School of Medicine, University of Vermont, Burlington, VT 05405, USA; 10 Department of Molecular Biology and Adelson, School of Medicine, Ariel University, Ariel 40700, Israel; 11 Department of Family Medicine, Jefferson Health Northeast, Philadelphia, PA 08033, USA; 12 Division of Nutrigenomics, Victory Nutrition International, Lederach, PA 19438, USA; 13 Department of Pharmacy and Health Sciences, College of Pharmacy and Health Sciences, Texas Southern University, Houston, TX 77004, USA; 14 Department of Psychiatry, Harvard Medical School, Boston, MA 02139, USA; 15 Department of Psychology & Behavioral Neuropharmacology and Neuroimaging Laboratory on Addictions (BNNLA), Research Institute on Addictions, University at Buffalo, Buffalo, NY 14203, USA; 16 Department of Psychiatry, South Texas Veteran Health Care System, Audie L. Murphy Memorial VA Hospital, Long School of Medicine, University of Texas Medical Center, San Antonio, TX 78229, USA; 17 Future Biologics, Lawrenceville, GA 30043, USA; 18 Department of Psychiatry, Washington University School of Medicine, St. Louis, MO 63110, USA

**Keywords:** Intermittent Explosive Disorder (IED) in Adolescence, GARS, Dopamine homeostasis, Reward Deficiency Syndrome (RDS), Dopamine D2 receptor gene polymorphism (*DRD2*), Dopamine Transporter Gene (*DAT1*)

## Abstract

Scientific studies have provided evidence that there is a relationship between violent and aggressive behaviors and addictions. Genes involved with the reward system, specifically the brain reward cascade (BRC), appear to be associated with various addictions and impulsive, aggressive, and violent behaviors. In our previous research, we examined the Taq A1 allele (variant D2 dopamine receptor gene) and the DAT-40 base repeat (a variant of the dopamine transporter gene) in 11 Caucasian boys at the Brown School in San Marcus, Texas, diagnosed with intermittent explosive disorder. Thirty supernormal controls were screened to exclude several reward–deficit behaviors, including pathological violence, and genotyped for the *DRD2* gene. Additionally, 91 controls were screened to exclude ADHD, pathological violence, alcoholism, drug dependence, and tobacco abuse, and their results were compared with *DAT1* genotype results. In the schoolboys vs. supercontrols, there was a significant association with the D2 variant and a trend with the dopamine transporter variant. Results support our hypothesis and the involvement of at least two gene risk alleles with adolescent violent/aggressive behaviors. This study and the research presented in this paper suggest that violent/aggressive behaviors are associated with a greater risk of addiction, mediated via various genes linked to the BRC. This review provides a contributory analysis of how gene polymorphisms, especially those related to the brain reward circuitry, are associated with violent behaviors.

## 1. Introduction

### The Issue

There is evidence that unwanted explosive, intermittently violent, and aggressive behaviors are associated with several reward genes related to dopaminergic function [1]. Violence and aggression pose a major clinical challenge to mental health care providers and represent a significant public health concern [2]. It is well-known that the aggression phenotype may represent an array of polymorphic genetic antecedents, differential neuroanatomy, altered regional volumes, and aberrant interregional connectivity [3]. Understanding the neurogenetics and neurobiology of the brain reward circuitry (i.e., serotonergic, cannabinoidergic, opioidergic, GABAergic, glutaminergic, and dopaminergic, etc.) provides clinically relevant targets for future therapeutic intervention, including early identification of aggressive behavioral risk. It is noteworthy that disciplinary and legal difficulties stemming from violent or aggressive behavior have reached epidemic proportions among our youth. Roughly 8% of adults and 17% of adolescents report a pattern of recurrent aggressive outbursts within one year. Such individuals are much more impulsive and aggressive than nonaggressive controls. They are also more likely to carry and use weapons, threaten others, engage in intimate partner assault, and be arrested by law enforcement [4]. Accordingly, the impact of aggressive and violent individuals begets significant economic and social costs to society. Moreover, individuals who display episodic rage attacks are reluctant to seek treatment, especially if the episodes are accompanied by physical violence [5]. There is also a recent emergence of new media technology and its subsequent increased use and access by adolescents (e.g., computers for internet access, cell phones). This technological explosion offers potential benefits as well as risks [6]. One area of concern for adolescents is that the risk of being subjected to aggression perpetrated by peers through these mediums is rising.

## 2. Substance Use Disorder (SUD) and Intermittent Explosive Disorder (IED)

According to Puhalla et al. [7], intermittent explosive disorder (IED) is the only psychiatric diagnosis for which the primary symptom is affective aggression. We know that an alcohol use disorder (AUD) diagnosis and a history of childhood abuse increase the likelihood of developing IED. Furthermore, an association exists between increased general aggression, including aggression while intoxicated, and both AUD and childhood abuse [7]. An investigation by Puhalla et al. [7] revealed that childhood abuse, but not AUD status, predicted IED status. Puhalla et al. showed that IED, aggression frequency, AUD, and childhood abuse were all independently associated with overall aggression; however, only individuals with IED displayed increased intoxicated aggression related to the degree of AUD severity [7]. Therefore, genetic vulnerability could be one possible neurobiological basis for this type of behavior [8]. For example, a history of childhood abuse could enhance the frequency of engaging in overall aggression and developing IED, which may increase the association between SUD severity and well-known intoxicated aggression.

It is essential to realize that aggression, rage, violence, alcohol, and drugs are all connected [9]. According to Alcoholics Anonymous terminology, anger is connected to relapse [10,11]. Clinically, violent outbursts and rage, for example, could be signs of delusional schizophrenia, bipolar disorder, posttraumatic stress disorder, antisocial, borderline, and paranoid character disturbances, and attention deficit disorder [12]. In terms of psychopharmacological mechanisms, the toxic effects of stimulant drugs like methamphetamine and cocaine induce hyperarousal and anxiety. Coinciding with this, the leading cause of death among stimulant drug addicts results from abuse and violence [13,14].

## 3. Neurogenetics of Intermittent Explosive Disorder (IED) and Violent Aggressive Behaviors

The origin of human violence has been an issue of significant concern for centuries [15,16]. Of genuine interest, violent behavior and possibly being born a “natural killer”, for example, raise questions related to the nature/nurture conundrum. We are aware that the phenotype of IED is characterized by inborn genetic polymorphisms as well as the impact of epigenetics. Several investigations have identified specific DNA polymorphisms that augment the risk for violent and antisocial behaviors [17]. There is evidence from behavioral genetics supporting the conclusion that genetic contributions are responsible for significant variance in antisocial personality and violent behavior.

Accordingly, Fergurson [17], in a meta-analysis, revealed that genetic influences could account for 56% of the variance in antisocial behavior, with 31% due to unique non-genetic influences and 11% due to shared non-genetic influences. Ferguson suggests that the resultant data reflect a relationship to evolutionary psychological theory. Evidence from behavioral genetics supports the conclusion that a significant amount of the variance in antisocial personality and behavior (APB) is due to genetic contributions. Many scientific fields, such as psychology, medicine, and criminal justice, struggle to incorporate this information with preexisting paradigms that focused exclusively on the external or learned etiology of antisocial behavior. Ferguson presents a meta-analytic review of behavioral genetic etiological studies of APB. Results indicated that 56% of the variance in APB can be explained through genetic influences, with 11% due to shared non-genetic influences and 31% due to unique non-genetic influences [17].

The consensus of the current literature provides evidence from twin, family, and adoption studies and makes a case for the significance of genetic factors in the nascence of aggression from childhood through adulthood [18]. Furthermore, data from adoption studies show that some environmental conditions (epigenetics) interact with genetic factors in a manner that suggests that both genetic and environmental factors must be present for aggressivity (violence) to develop [19]. One study revealed an association between the CDH13 gene (Cadherin 13, which codes for neuronal membrane adhesion protein) and a monoamine oxidase-A (MAO-A) low-activity genotype (which contributes to a low dopamine turnover rate) and highly violent behavior among two independent cohorts of Finnish prisoners. However, no significant signal was detected for either CDH13 or MAO-A in non-violent offenders in this study cohort, signifying that findings were limited to violent offending and not primarily the result of antisocial personality disorder or substance abuse [19]. The authors suggest that, for example, both low neuronal membrane dysfunction and monoamine metabolism are possible factors in the etiology of extremely violent criminal behavior and suggest that about 5–10% of all severe violent crimes in Finland can be attributed to the MAO-A and CDH13 genotypes.

Neuronal membrane potential is of fundamental importance for the mechanistic understanding of brain function. Whole-cell recordings can be combined with two-photon microscopy to target fluorescently labeled neurons, revealing cell-type-specific membrane potential dynamics of retrogradely or genetically labeled neurons. Dual whole-cell recordings reveal behavioral modulation of membrane potential synchrony and properties of synaptic transmission in vivo. Optogenetic manipulations are also readily integrated with whole-cell recordings, providing detailed information about the effect of specific perturbations on the membrane potential of diverse types of neurons.

In addition, specific genes related to the underlying biochemical mechanisms associated with aggressivity in animals are similar to the configuration of similar physiologic mechanisms in humans [20]. Therefore, in the following section, we will provide a snapshot of the various roles of “reward” genes and associated addiction risk polymorphisms in IED, primarily in humans.

**Figure 1:**
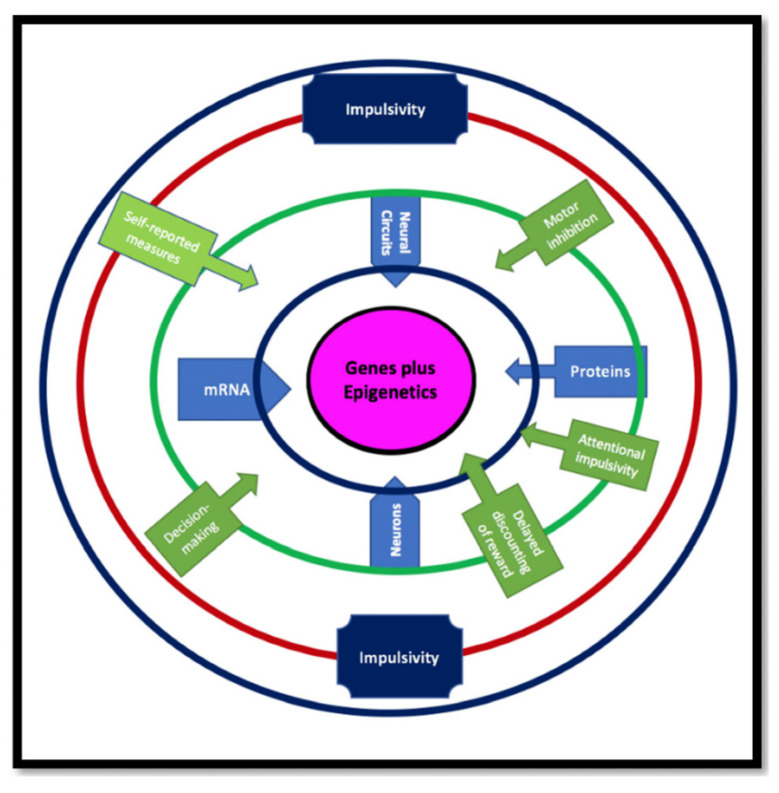
Impulsivity is a hereditary, disease-associated trait that may be evolutionarily beneficial as an endophenotype for genetic innovation resulting in behavioral adaptation to a challenging environment. More specifically, impulsivity is not a singular construct but a complex trait. Multiple laboratory behavioral tasks and self-report measures are used to assess aspects of impulsivity. Different neural circuits and genes are impacted by epigenetics, which also has pleiotropic effects on behaviors that modulate impulsivity.

Furthermore, we suggest that defining different forms of impulsivity/impulsive behaviors related to these genes might evolve our understanding of the neurobiological basis of disorders for which impulsivity is a component. These disorders include IED and other aggressive and violent behaviors associated with other psychopathologies. Finally, we combine all these interacting factors with impulsivity into a model shown in Figure 1.

## 4. Specific Reward Genes and IED

In 1989, Blum and Kozlowski published their initial concept of the brain reward cascade (BRC) implicated in various addictions and other behaviors [21]. In previously published works from our laboratory, we proposed a BRC schematic at mesolimbic sites as described in Figure 1. Based on the overlapping evidence previously given in this paper, this cascade appears to be involved in IED and violent and aggressive behaviors [22].

Moreover, our laboratory has developed United States and foreign patents related to the Genetic Addiction Risk Severity (GARS) Test [23]. Table 1 displays the associated various risk alleles from the BRC that can be measured.

Evaluation of pain clinic patients with the GARS test and the addiction severity index-media version V revealed significant GARS scores. Specifically, scores of seven or greater were associated with alcohol, whereas scores of four or greater were associated with other drugs [24].

## 5. Specific Genes from the BRC/GARS: Linking Them with IED and Aggressive, Violent, and Impulsive Behaviors

### 5.1. Dopamine D1 Receptor

A recent search (10 October 2021) on the PubMed database using the term “Intermittent Explosive Disorder” (IED) and “Dopamine D1 Receptor” (*DRD1*; rs4532) located on chromosome 5 resulted in no listings. However, additional searching related to impulse control behaviors/disorders (ICB/ICD) did result in many studies. ICB/ICD are characterized by an inability to resist urges that result in an individual engaging in behaviors that are harmful to themselves or others [25]. An abundance of research has been focused on polymorphisms of the *DRD1* (particularly rs4532) concerning its role in various neuropsychiatric disorders [26]. As a result, associations between the *DRD1* Rs4532 polymorphism and several mental illnesses have been uncovered, including nicotine addiction [27], bipolar disorder [28], and ADHD [29]. Genotyping of several dopaminergic polymorphisms detected pronounced differences between variant and wild-type alleles using a high-resolution melt (HRM).

Specifically, associations between variants of *DRD1* rs4532 (OR = 21.33; 95% CI, 1.97–230.64; *p* = 0.0024) and an increased risk of developing ICB were observed in a cohort of Parkinson patients. In addition, using the catalyst model of aggression as guidance revealed risk alleles that were hypothesized to be associated with intimate partner violence (IPV) in the presence of financial hardship [30].

### 5.2. DRD2/ANKK1

The rs1800497 variant in *DRD2*/ANKK1 has been implicated in a reduction in receptor density in the striatum [31]. The rs1800497 variant in *DRD2*/ANKK1 results in significant protein structure modifications that manifest in reduced expression of striatal receptors (possibly due to rs1800497 changing glutamic acid to lysine). Self-reported measures of impulsivity are approximately 45% heritable, as demonstrated by twin studies [32]. The most frequent association between addiction and the *DRD2*/ANKK1 gene is found with the rs1800497 polymorphism [33]. In fact, the *DRD2* gene is responsible for the most consistent findings relating to the genetics of impulsivity as well. Response inhibition testing has implicated the *DRD2* A1 allele with impulsive behavior among healthy young adults [34,35]. Another study [36] conducted with a young adult population uncovered an association between impulsive self-damaging behaviors (assessed using borderline symptoms) and the *DRD2* gene (A1 and B1 alleles). Impulsivity in healthy individuals was also correlated with other *DRD2* SNPs. A relationship between the C/C genotype of the *DRD2* C957T polymorphism and higher reward responsiveness after a psychological stressor was also detected [34]. In a neuroimaging study, the *DRD2*-141C Del carriers displayed greater reward-related ventral striatum reactivity, which was associated with self-reported impulsivity [37]. A study by Zainal Abidin et al. [26] reported an association between an increased risk of developing ICB and the *DRD2*/ANKK1 rs1800497 (OR = 3.77; 95% CI, 1.38–10.30; *p* = 0.0044) in a cohort of patients with Parkinson’s disease.

A critical achievement that illustrates these concepts is a study by Caspi et al. [38] that reported the most extensively cited measured Gene X Environment (GXE) interaction in predicting antisocial and violent behavior. Furthermore, Boardman et al. [39] reported that in the presence of low family violence, the carriers of two copies of the A1 allele variant of *DRD2* are significantly more likely than those with one or no copies of the A1 allele to engage in serious delinquency. However, when those same individuals perceive a high level of family closeness, they are significantly less likely than those with no copies of the A1 allele to report higher delinquency.

It is noteworthy that when African American females carry at least one A1 allele of *DRD2*, they are more likely to experience violent victimization and have higher levels of depressive symptoms.

Results also show that *DRD2* imparts a significant independent effect on depressive symptoms in female and male African-Americans [40] and African American children [41]. It is moderately established, albeit, with some controversy, that lower-functioning dopamine systems motivate individuals to seek rewards from external sources such as illicit drugs and other risky experiences. Along these lines, work from Chester et al. [42] revealed that amongst Caucasian males and females, *DRD2* profiles were associated with increased sensation-seeking, which then predicted increased aggression. Chester et al. [42] suggested that decreased dopaminergic functioning elevates an individual’s risk for violence because it motivates them to experience the hedonically rewarding qualities of aggression. In addition, a study by Zai et al. demonstrated that the occurrence of at least one copy of the G allele for the *DRD2* A-241G polymorphism (genotypic *p* = 0.02; allelic *p* = 0.01) was significantly associated with aggressive children [43]. Additionally, the *DRD2* rs1079598 CC-genotype was overrepresented in aggressive children compared to controls (genotype *p* = 0.04). Amongst aggressive children, the T.T. genotype (*p* = 0.01) and the *DRD2* TaqIA T allele (*p* = 0.01) were also found to be significantly overrepresented.

### 5.3. Dopamine D3 Receptor Gene

Importantly, drug abuse and violence are immensely destructive phenomena found worldwide. From 105 postmortem cases, a significant genetic difference has been reported for SNP rs6280 from the *DRD3* gene that displayed a significant association, with genotypes T/C and C/C being more frequent in drug users (OR = 4.96; 95% CI = 1.07–23.02; *p* = 0.04), including cocaine and risky/violent behaviors [44].

### 5.4. Dopamine 4 Receptor Gene

Variable-number tandem repeat (VNTR) polymorphisms of the *DRD4* gene were investigated in Chechen and Russian men convicted of crimes. The *DRD4* long alleles were found to be more frequent in the men convicted of felonies, a finding similar to a cohort of mixed martial art (MMA) fighters that lacked a criminal record in both paternal lines [45]. Furthermore, increased vulnerability for impulsive and antisocial behavior in response to aversive environmental conditions has been associated with the *DRD4* (dopamine D4 receptor) VNTR 1-11 [46]. Using data from the National Longitudinal Study of Adolescent Health, Daigle [47] reported that the 7R allele of the *DRD4* gene distinguishes individuals who have been victimized multiple times from those who have been victimized once. According to Buchmann et al. [48], carriers of the *DRD4* seven-repeat allele demonstrated more aggression in adulthood (*p* = 0.032) under conditions of increased maternal stress, which extended earlier observations regarding childhood antisocial behavior. In Marsman et al. [49], parental overprotection and rejection predicted higher levels of externalizing behavior problems (EBP), while lower levels of EBP were predicted by parental emotional warmth. They did find interaction effects with familial loading of externalizing behavior problems (FLE) and the *DRD4* (specifically a 4-repeat allele). In more detail, the predictive effect of parental rejection was only observed in adolescents from low-FLE families, and the predictive effect of parental overprotection was more robust in adolescents not carrying the *DRD4* 4-repeat allele. Nobile et al. [50] found that the *DRD4* long allele is associated with higher aggressive behavior scores in Italian preadolescents. Previous research has reported associations between the externalization of problems and aggression in children to either harsh and insensitive parenting or *DRD4* polymorphisms. In the study by Bakermans-Kranenburg and van Ijzendoorn [51], they determined maternal insensitivity was correlated with externalizing (oppositional, aggressive) behaviors, but only with the occurrence of the *DRD4* 7-repeat polymorphism. Children who were positive for the 7-repeat allele were six times more likely to have externalized behavior when exposed to insensitive care than children without these genetic and environmental antecedents.

### 5.5. COMT

There is some evidence that both environmental and genetic factors influence an individual’s propensity for aggression. Both the functional polymorphism catechol-O-methyltransferase Val158Met (*COMT*) and childhood experiences of adversity have been implicated in aggression and aggression traits [52]. Hygen et al. showed that childhood serious life events and the *COMT* genotype had a significant interactive effect on childhood serious life events [52].

Specifically, Val homozygote children who had endured many serious life events displayed more aggression (*p* = 0.02) than did their Met-carrying peers. Of great interest, Brennan et al. [53] found the *COMT* Val108/158Met polymorphism (rs4680) substantially interacted with maternal cigarette smoking during pregnancy to predict aggressive youth behaviors at ages 15 and 20. Other work by Wang et al. [54] found that the Val/Met heterozygote and the Val/Val homozygote carriers displayed differences in aggressive motivation and feelings of hostility under conditions of inclusion versus exclusion. However, the differences were more prominent for Val/Met allele carriers, as expected in terms of the genetic phenomena heterosis [55].

Molecular heterosis occurs when subjects heterozygous for a specific genetic polymorphism show a significantly greater effect (positive heterosis) or lesser effect (negative heterosis) for a quantitative or dichotomous trait than subjects homozygous for either allele. Commings reviewed the accumulating evidence that molecular heterosis is common in humans and may occur in up to 50% of all gene associations. A number of examples are reviewed, including those for the following genes: ADRA2C, C3 complement, *DRD1*, *DRD2*, *DRD3*, *DRD4*, ESR1, HP, HBB, HLA-DR DQ, HTR2A, properdin B, SLC6A4, PNMT, and secretor [55].

It is known that childhood maltreatment and cannabis use are independent risk factors that increase the probability of experiencing psychotic symptoms [56]. Vinkers et al. [56] uncovered a significant three-way interaction between the *COMT* genotype, cannabis use, childhood maltreatment, and [rs4680] (*p* = 0.006). In fact, Val-homozygous individuals exhibited increased psychotic experiences after exposure to both childhood maltreatment and cannabis use, compared to Met-homozygous individuals and Met-heterozygous individuals.

### 5.6. Mu-Opioid Receptor [OPRM1]

Opioids regulate mesolimbic dopaminergic pathways in the VTA via activation of μ-opioid receptors on secondary interneurons, which cause hyperpolarization and inhibition of GABA release on primary neurons (the dopaminergic output neurons), and consequently increased DA release [57]. The Colorado Center for Antisocial Drug Dependence (CADD) has been using several research designs and strategies to study the genetic basis for antisocial drug dependence in adolescents. They found that the mu-opioid receptor gene (*OPRM1*) rs495491 significantly emerged as a plausible candidate for a role in antisocial drug dependence after gene-based permutation tests with a *p*-value of *p* < 0.006 of this SNP (odds ratio 1.47) [58]. Interestingly, opiates have been related to aggression, specifically self-directed aggression. Illicit opioid use, misuse, and intoxication can result in violence, while a decrease in opioid availability can result in OUD victims acting violently in order to obtain supplies [59]. Moreover, self-injurious behavior has been associated with enhanced metenkephalin [60], while opiate antagonists typically diminish self-injurious behavior [61]. Attenuated Cerebral Spinal Fluid (CSF) endogenous opioid concentrations have been associated with self-injurious behaviors in patients with borderline personality disorder [62]. It is known that decreased opioids may be related to increased rejection sensitivity and abandonment/separation distress and may heighten the likelihood of aggressive behavior [63,64]. It is essential to realize that attenuated presynaptic opiate activity may upregulate postsynaptic µ-opioid receptors, and thus dramatic relief of pain may result when opiates are released in the context of self-injurious behavior [65].

Polymorphisms in *OPRM1* are the primary candidate sources of clinical variability in opioid therapy. Apart from the 118 A > G single nucleotide polymorphism, nothing is known about the role of *OPRM1* mutations in opioid therapy [66]. In a study by Lötsch et al., the influence of the *OPRM1* mutations on opioid pharmacodynamics was assessed using pooled data from 31 healthy volunteers obtained in previous studies with available plasma concentrations and pupil diameters after intravenous administration of morphine or morphine-6-glucuronide (M6G) [66]. A total of 24 candidate ORPM1 mutations were screened for, and those found at an allelic frequency of at least 5% in the 31 subjects were analyzed for functional consequences using population pharmacokinetic-pharmacodynamic modeling of the miotic effects of the opioids as a reliable and sensitive surrogate parameter of the central nervous system opioid effects. Polymorphisms with an allelic frequency of > or = 5% (*n* = 310) were 118 A > G in exon 1 (11.5%), IVS2-31 G > A (8.9%), and IVS2-691 C > G (44.5%) SNPs in intron 2. The 118 A > G SNP significantly increased the values of EC50 by a factor of more than 2 (non-mutated: EC50, morphine = 30 nmol/L, EC50, M6G = 750 nmol/L, 118 G carriers: EC50, morphine = 66 nmol/L, EC50, M6G = 1650 nmol/L), whereas the IVS2-691 C > G SNP had no effect. Based on morphine and M6G, the present analysis encourages focusing on the 118 A > G SNP when investigating the role of *OPRM1* mutations in the activity of opioid analgesics. Other *OPRM1* mutations are probably less important, either owing to low allelic frequency or due to poor indications for functional consequences. This applies to opioid potency in the context of opioid therapy but not to pain processing or substance addiction, in which opioid receptors are involved but other or additional *OPRM1* mutations may be important.

Other work by Cimino et al. [67] found that mothers and children who carried the G allele (G/G + A/G genotypes) were more likely to have an insecure attachment style. Children with the G-allele scored higher than homozygous A/A children on the withdrawal and conduct problems subscales in the clinical sample. In fact, mothers with the G-allele displayed elevated interpersonal sensitivity, hostility, depression, paranoid ideation, and hostility and provided less care than mothers with the A/A allele. Further interest resides in the fact that individuals with the G allele tend to experience more social pain [68,69] and increased emotional dysregulation and neural activation as a consequence of social rejection. In contrast to A/A homozygotes, individuals with the G-allele also demonstrate higher levels of rejection sensitivity, behavioral retraction to angry faces, and high levels of fearful attachment despite the quality of their early maternal care [70,71,72]. This demonstrates that the A118G-genotype modulates the effects of early maternal care on adult attachment style.

### 5.7. Dopamine Transporter (DAT1)

Dopamine transporter DAT-1 (SLC6A3) is a critical dopaminergic system gene that modulates dopamine signaling and reuptake and may contribute to several psychiatric disorders, such as antisocial behaviors and traits. Convicted murderers were more likely than controls to be carriers of the 9R allele of thee DAT-1 VNTR polymorphism for either one or two risk alleles (OR = 1.49 and 3.99, respectively, *p* = 0.003). Different genetic inheritance models were used to validate the plausible association between the DAT-1 9R allele polymorphism and the “murderer” phenotype. Furthermore, this identified phenotype was associated with the combined haplotype of the *DRD2* and 9R-A2 of DAT-1 genes. Moreover, parental marital complications and responses to verbal abuse were correlated to the 9R allele of DAT-1. The results allude to the role of the 9R allele in contributing to criminal propensity in convicted murderers of Pakistani origin [73]. Further, carriers of the high-risk *DAT1* alleles were more likely to commit IPV in the presence of financial stressors than the individuals carrying low-risk alleles [30]. Investigations by Fine et al. [74] have uncovered interactions stemming from the effects of dopaminergic phenotypes and school attachment on delinquency. Negative and positive school environments conferred different effects on individuals carrying the *DRD2*-A1 allele. Whereas individuals carrying the *DAT1*-10R allele fared better in positive environments, they fared the same as 9R homozygotes in moderate and poorer environments. It is also notable that Young et al. [75] demonstrated that the *DAT1* 9-repeat variant conferred a significant risk for externalizing behavior at ages four (*p* = 0.001) and seven years (*p* = 0.02). Data from twin studies present evidence that genetic factors may contribute to adolescent-onset or adolescent-limited antisocial behavior. Burt and Mikolajewski [76] presented evidence that adolescent antisocial behavior (ASB) was associated with *DAT1*. However, these associations were only found in a nonaggressive, rule-breaking subset of ASB, and they failed to reach statistical significance in the context of physical aggression. Finally, as mentioned in the D1 dopamine receptor gene section, as predicted, individuals with high-risk *DAT1* alleles were shown to be more likely to commit IPV in the presence of financial hardship than individuals with low-risk *DAT1* alleles [30].

### 5.8. Monoamine Oxidase-A (MAO-A)

A variable number of tandem repeats (VNTR) of the monoamine oxidase A (MAO-A) gene promoter have been correlated with the expression of antisocial behavior in hostile or stressful environments. Uršič et al. [77] provided some evidence to support the association of the MAO-A polymorphism with suicide. Specifically, Uršič et al. [77] demonstrated a trend towards the 3R allele and suicide and associated the 3R allele with the non-violent suicide method on stratified data (20 suicide victims). Beaver et al. [78] reported that the decreased function of the MAO-A allele augmented the risk of joining a gang and increased the odds that an individual would use a weapon during a fight, an observation that was only valid for males, not females. The relationship between the low MAO-A activity allele and increased chances of using a weapon during a fight stayed relevant even when comparing gang members; gang members without the low-activity MAO-A allele were less likely to utilize weapons. In agreement with the latter findings, Kolla and Vinette [79] also found that the low-activity MAO-A variable nucleotide tandem repeat genetic polymorphism demonstrated strong relationships with large samples of violent offenders, many of whom had an antisocial personality disorder (APD). These same authors, Kolla et al. [80], indicate that low-activity monoamine oxidase-A genotype may affect cortico-striatal connectivity in APD. They also suggest that this postulated connectivity may also contribute to proactive aggression in a genotype-specific manner, conceivably resulting from the enhanced dopaminergic activity.

Along similar lines, Gera et al. [81] dissected the low-activity of the MAO-A genotype from the high-activity genotype. Specifically, they found the prevalence of the low-activity 3-repeat allele was more common in violent offenders who were heroin addicts than in addicts without antisocial behavior (34.6 vs. 15.4%; *p* < 0.03) and controls (18.9%; *p* < 0.05). In contrast, high-activity 4-repeat allele frequency was more prevalent among individuals with no antisocial behavior than among individuals that were antisocial-aggressive heroin-dependent (76.9 vs. 55.8%; *p* < 0.02). Also, heroin addicts with the low-activity 3-repeat allele scored significantly higher in the Buss Durkee Hostility Inventory (BDHI) on the irritability, suspiciousness, and resentment subscales than individuals with high-activity alleles. It was also found [82] that decreased MAO-A function within the cortical and subcortical brain regions manifested in increased self-reported aggression and contributed to over one-third of the variability. In contrast, others found the increased activity of the MOA-A allele to be associated with proactive aggression among violent offenders with antisocial personality disorder [83]. Huang et al. [84] found that the decreased expression allele was correlated with high impulsivity in males with a known history of abuse (before 15 years); the same was not true in females. This known polymorphism may potentially be a marker for impulsivity, which may also play a role in the risk of abuse. However, Ni et al. [85] reported that among patients with borderline personality disorder, the high-activity VNTR alleles were more common (chi = 4.696, *p* = 0.03), and the low-activity haplotype (*X*^2^ = 5.089, *p* = 0.02) was less common. To help us understand dopaminergic dynamics, Schlüter et al. [86] found that the *MAOA*-High group displayed higher aggression and augmented dopamine release after watching a violent movie. However, the violent film reduced aggression without causing consistent increases in dopamine release in subjects with low-activity MAO-A. Accordingly, these results suggest a plausible influence of the MAO-A-promoter polymorphism on the neurobiological regulation of aggressive behavior. However, the notion that low MAO-A promotes aggression via the singular effect of augmented dopamine is not supported by data. Indeed, many studies support the role of the MAO-A gene as a prominent genetic determinant for criminal violence [87,88,89,90,91,92,93,94,95,96,97,98,99,100,101].

### 5.9. Serotonin Transporter Gene (5-HTTLPR)

Tielbeek et al. [102] performed a meta-analysis of eight studies consisting of 12 independent samples with a total of 7680 subjects; an effective sample of 6724 subjects was included. They found a significant association between environmental adversities and the 5-HTTLPR genotype on antisocial behavior. Tung and Lee [103] also found that in 2558 adolescents the 5-HTTLPR genotype significantly modulated the correlation of parental support with ASB membership. Specifically, individuals with the short allele demonstrated higher sensitivity to parental support when predicting late-onset trajectory; the long/long genotype served as a plausible “plasticity genotype” for the adolescent-peak trajectory group. Others [104,105] show the prevalence of the S allele and the S.S. genotype to be higher among violent suicide attempters than among subjects in the control group. The S.S. genotype odds ratio compared to the L.L. genotype was 3.63 (95% CI (1.27–10.40). This difference implies that an alteration in the expression of the 5-HT encoding transporter gene may play a role in violent suicidal behavior. Retz et al. [106] found that an excess of the S/S genotype and the short (S) alleles in Caucasian male patients was characterized by reoccurring physical violent behavior and explicated 5% of the genetic variance of violent behavior. From clinical research, Gorodetsky et al. [107] differentiated influential variables on self-directed aggression among groups of similar predictor variables within the expressed behavioral domain. 5-HTTLPR expressed an independent gene dose-dependent effect. Furthermore, Hallikainen et al. [108] found the S allele prevalence was greater in Type II alcoholics than in Type I alcoholics (*X*^2^ = 4.86, *p* = 0.028) and healthy controls (*X*^2^ = 8.24, *p* = 0.004). This work suggests an association between the 5-HTT ‘S’ promoter polymorphism and an increased risk for early-onset alcoholism related to antisocial personality disorder and impulsive, repetitive violent behavior. Chinese investigators [109] also found that S-allele positive subjects were significantly greater in the criminal group than among the controls (*p* = 0.006). In other work, [110] suicide-related trait measure analyses of variance conducted on the three genotypes established a significant difference in violence measures among patients with the L.L. and L.S. genotypes (9.50 ± 4.04 vs. 5.36 ± 4.03; *p* = 0.029). This study implies that the 5-HTTLPR polymorphism may contribute to violent behavior in this population. In addition, it was found that maltreatment in childhood adds to the risk of ASPD, and preliminary evidence suggests the 5-HTTLPR genotype moderates the effects in women. It is known that the 5-HTTLPR polymorphism has been correlated with both aggression/hostility and depression [111]. Specifically, Gonda et al. [111] found a significant correlation with many hostile traits. The interaction between the two primary effects was also significant in the context of several subscales. Post hoc analyses resulted in a significant correlation between the S allele (only in the depressed group) and the BDHI subscales.

The second-highest reason for death among youth worldwide is suicidal behavior, and it is also the tenth-leading cause of death among all age groups. Inherited genetic differences have a part in suicidality, with heritability ranging from 30 to 55% [112]. A literature search revealed 1186 articles; among these, Fanelli and Serretti [112] identified 45 pertinent case-control studies (15,341 subjects). Low-expressing alleles (S + LG) were correlated with an increased risk of violent suicide attempts (OR = 1.44, C.I.: 1.17–1.78, *p* = 0.0007). It is noteworthy that studies have not provided clear evidence concerning the genetic background of suicidal predisposition. However, the associations between polymorphisms of the 5-HTTLPR genes and violent suicidal behavior reveal the fewest inconsistencies [113,114,115].

### 5.10. GABA (A) Receptor Gene (GABRB3)

A word search in the PubMed database utilizing terms in a Boolean search including various combinations of “violent behavior”, “crime”, “criminal justice system”, “suicide”, “IED”, “aggression”, and “bullying” with GABRB3 resulted in no listings.

### 5.11. Original Dopaminergic Candidate Association Study in Adolescent IED

Previously, Blum’s group hypothesized that polymorphisms in the dopaminergic system might contribute to pathological aggression in adolescents [116]. Although multiple neurotransmitter systems are likely involved, one major critical pathway should involve the dopaminergic system. In addition, a more profound grasp of the neurobiological foundations of aggression has led to pharmacological treatments for such behaviors. As discussed above, the primary biological networks that contribute to reward neurotransmitters include opioid peptides, serotonin, catecholamines (norepinephrine and dopamine), and γ-aminobutyric acid.

This research employed a small pilot study involving 11 Caucasian boys, aged 13–19, who were diagnosed with IED and violent/aggressive behaviors in adolescence and attended the Brown School, a residential school facility in San Marcus, Texas. This study received IRB approval from the Path Foundation in New York, NY. Each patient signed an approved consent form. Each patient was genotyped for the *DRD2* Taq A1 (rs1800497) and the *DAT1* (40BP repeat VNTR) polymorphisms [116,117].

Results from this study revealed a correlation between *DRD2* Taq A1 (rs1800497) and *DAT1* (40 B.P. repeat VNTR) polymorphisms and IED. In addition, the subjects were diagnosed with IED (characterized by pathological violence or impulsive and aggressive violent behavior). Furthermore, 30 supernormal controls were evaluated and screened for several reward deficit behaviors, including pathological violence; only 3.3 percent of them carried the *DRD2* Taq A1 (rs1800497). Moreover, another 91 controls were also screened to exclude pathological violence, ADHD, drug dependence, alcoholism, obesity, and tobacco use. This second set of controls was used to evaluate the absence or presence of the *DAT1* (40BP repeat VNTR) and polymorphisms in the cases and controls. We found in this small pilot some interesting genetic correlations. When the *DRD2* A1 (A1/A1 or A1/A2) genotype was compared in these subjects to the super controls, a robust significant association was observed whereby *X*^2^ = 14.9, df = 1, and *p* = 0.0001, indicating that when highly screened controls are utilized, carriers of the *DRD2* A1 allele reveal a clear association toward violent behaviors. However, a similar trend was found with the *DAT1* 480 bp 10/10 genotype compared to controls (*X*^2^ = 2.82, df = 1, and *p* = 0.093). Most importantly, when the 9/10 genotype was compared with these controls, a significant association was observed (*X*^2^ = 14.31, df = 1, *p* = 0.00006), indicating that when highly screened controls are utilized, carriers of the *DAT1* 480 bp allele reveal a clear association toward violent behaviors. It is noteworthy that the data relevant to the “super controls” and the initial data have been published elsewhere.

A blinded clinical trial identified a positive association between adolescents’ pathological violence, *DAT1* polymorphism, and the *DRD2* gene. Consequently, this and other cited work suggest a function for both the DAT and *DRD2* genes in manifesting aggressive behavior. Furthermore, these initial data obtained in 2005, in terms of these two potential candidates, agree with previously mentioned studies in this perspective for the *DRD2* A1 allele [31,32,33,34,35,36,37,38,39,40,41,42,43] and the *DAT1* 9 R allele [30,73,74,75,76].

## 6. Violent/Aggressive Behaviors and Addiction Liability

It is important to note that we are trying to provide evidence for the link between aggressive and violent behaviors and the liability of addiction in this paper. Our previous work suggested that addiction, impulsivity, and chronic violence may cluster together within the reward deficiency syndrome (RDS) [118]. The connection between addiction and violence may indeed be impulsivity, as seen among substance users with multiple incarcerations [119] and gamblers who binge-drink and are involved in non-partner physical aggression [120]. Alcohol also appears to be correlated with violence in many studies: among adolescents [121]; with alcohol ingestion being more likely to occur prior to violent incidents [122]; with changes in alcohol consumption modulating the degree of suppressed anger in those with extreme anger associated with violent behaviors [123]; among those admitted to emergency rooms with alcohol misuse who have a history of partner violence [124]; among university athletes who drink excessively and are violent both on and off the field, tied to masculinity [125]; and within the context of dating, alcohol use seems to mediate violence [126]. Methamphetamine addiction is also correlated with violence [127]. Gambling and substance abuse are also associated with violence [128]. Crack cocaine abusers were more likely to have an antisocial personality disorder and PTSD and were more likely to be both victims and perpetrators of violence [129,130]. Comorbid bipolar disorder and heroin addiction are correlated with violence as well [131]. In addition, various addictive disorders and substance abuse disorders are related to violence, including sexual abuse and domestic violence [132], interpersonal violence [133], and suicide [134]. Non-partner and partner violence are associated with drinking episodes, cocaine use, and depressive symptoms [135]. Crack cocaine and alcohol dependence are associated with aggression and violent behavior [136]. Finally, polysubstance use (including binge drinking, marijuana, other illicit drugs, and prescription stimulants) was associated with emergency room visits and a history of aggression and violent behaviors [137,138]. Finally, similar to this overview, Ahmadi and colleagues searched for studies for their review and determined that 15 out of the 19 studies they reviewed showed a significant correlation between various forms of aggression and addiction [139].

## 7. Dopaminergic Dysregulation (Surfeit or Deficit) Associated with Addiction and Violent Behavior

**Figure 2:**
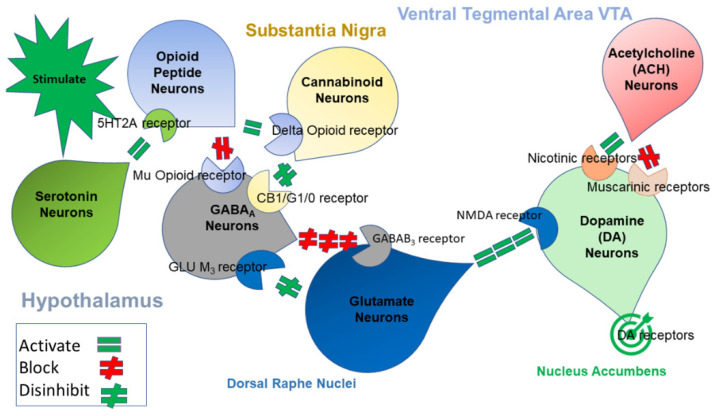
Illustrates the interaction of at least seven significant neurotransmitter-pathways involved in the brain reward cascade (BRC). In the hypothalamus, environmental stimulation results in the release of serotonin, which in turn, via 5HT2A receptors, activates (equal green sign) the subsequent release of opioid peptides from opioid peptide neurons, also in the hypothalamus. Then, the opioid peptides have two distinct effects, possibly via two different opioid receptors. One inhibits (red hash sign) through the mu-opioid receptor (possibly via enkephalin) and projects to the substantia nigra to GABAA neurons. Another stimulates (equal green sign) cannabinoid neurons (the anandamide and 2-arachidonoylglycerol, for example) through beta–endorphin linked delta receptors, which in turn inhibit GABAA receptors in the substantia nigra. Also, when activated, cannabinoids, primarily 2-archydonoglcerol, can indirectly disinhibit (red hash sign) GABAA receptors through activation of G1/0 coupled to CB1 receptors in the substantia nigra. In the dorsal raphe nuclei, glutamate neurons can indirectly disinhibit GABAA receptors in the substantia nigra by activating group III metabotropic glutamate (GLU M_3_) receptors (green hash). GABAA receptors, when stimulated, will in turn powerfully (red hash signs) inhibit ventral tegmental area (VTA) glutaminergic drive via GABAB 3 receptors. It is also possible that stimulation of ACh neurons at the nucleus accumbens can stimulate both muscarinic (red hash) and nicotinic (green hash) receptors. Finally, glutamate neurons in the VTA will project to dopamine neurons stimulating NMDA receptors (equal green sign) to preferentially release dopamine at the nucleus accumbens, shown as a green bullseye that indicates a euphoria or “wanting” response. The result is that when dopamine release is low (dopamine deficiency), it results in unhappiness, while general (healthy) happiness depends on the dopamine homeostatic tonic set point. (With permission from Blum et al. With permission from Blum et al. [139].).

**Figure 3:**
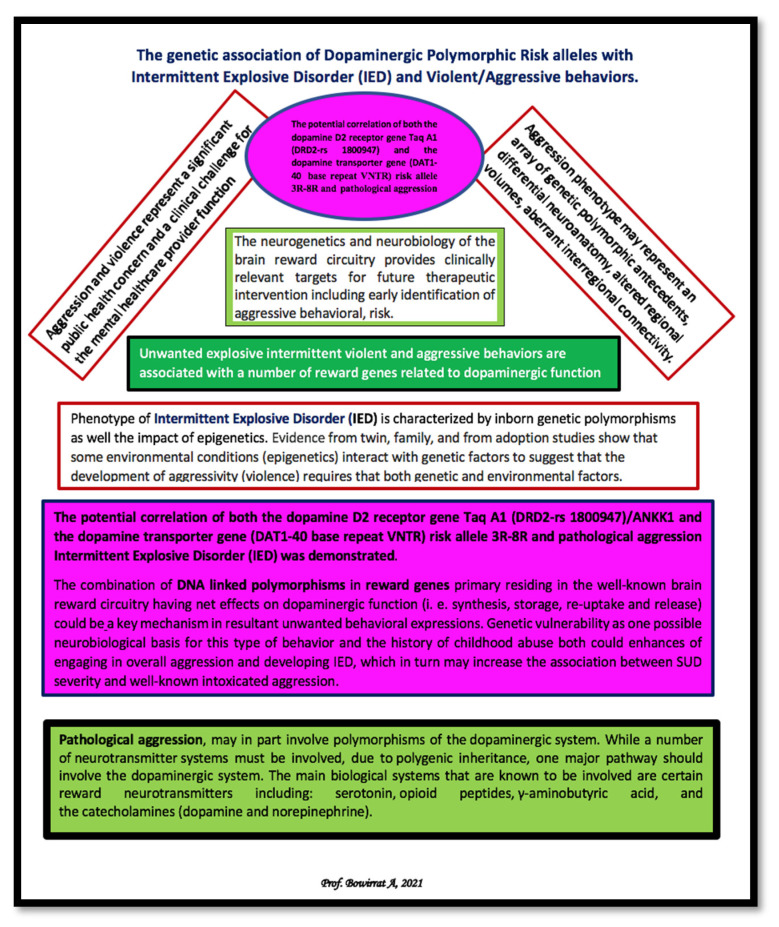
A schematic representing a summary of dopaminergic genetics with intermittent explosive disorder (IED), violent/aggressive behavior, and addiction.

We hypothesize that continued genetic research in this area will confirm positive associations with dopaminergic polymorphisms. The use of highly screened controls to eliminate addictive, impulsive, and compulsive behaviors (in both proband and family) may be of necessary consequence for our young population. The issue at hand is the present confusion related to whether the neurotransmitter genotypes that regulate downstream dopamine release and function at the BRC (Figure 2) are mixed in terms of surfeit or deficit in terms of reward processing. Evidence for both surfeit and/or deficit in reward processing seems unclear and requires additional research. To assist in understanding our model, developed by one of us (A.B.), the following schematic is provided (Figure 3) [139].

## 8. Conclusions

Following the seminal findings of our group in 1990 related to the association of the *DRD2* Taq A1 allele (located in exon 11 of the ANNKI gene) with severe alcoholism, 31 years later, along with an explosion of new genetic techniques (e.g., genome-wide association studies), genetic polymorphisms linked to extreme violence/aggression have been discovered. Possibly, the combination of DNA-linked polymorphisms in reward genes, which are primarily residing in the well-known brain reward circuitry and having net effects on dopaminergic function (i.e., synthesis, storage, reuptake, and release), could be a key mechanism in resultant unwanted behavioral expressions. However, the current literature data point towards an over-representation of the reward genes and associated risk alleles measured in the GARS test (see Table 1) in violent/aggressive behaviors. Interestingly, several specific risk alleles that load onto violent and aggressive behaviors are of evolutionary importance and an adaptive process linked to survival. We believe that this article provides a framework to encourage additional scientific exploration that couples these known polymorphisms as depicted herein with a genetic propensity to engage in, for example, domestic violent behaviors, which seem to be an inheritable legacy.

## Figures and Tables

**Table 1:** Represents the GARS SNPs and VNTRs (snapshot).

Gene	Polymorphism	Location	Risk Allele(s)
*DRD1*	rs4532	Chr 5	A
*DRD2*	rs1800497	Chr 11	A
*DRD3*	rs6280	Chr 3	C
*DRD4*	rs1800955	Chr 11	C
	48 bases Repeat	Chr 11, Exon 3	7R, 8R, 9R, 10R, 11R
*COMT*	rs4680	Chr 22	G
*OPRM1*	rs1799971	Chr 6	G
*DAT1*	40 bases Repeat	Chr 5, Exon 15	3R, 4R, 5R, 6R, 7R, 8R
*MAOA*	30 bases Repeat	Chr X, Promoter	3.5R, 4R
*Serotonin Transporter SLC6A4 (5-HTTLPR)*	43 bases Repeat plus rs25531	Chr 17	LG, S
*GABA(A) Receptor, Alpha-3 GABRB3*	CA-Repeat DNR	Chr 15 (downstream)	181

Abbreviations: Dopamine receptor D1 (*DRD1*), dopamine receptor D2 (*DRD2*), dopamine receptor D3 (*DRD3*), dopamine receptor D4 (*DRD4*), catecholamine-methyltransferase (*COMT*), opioid receptor mu 1 (*OPRM1*), dopamine transporter (*DAT1*), monoamine oxidase A (*MAOA*), serotonin-transporter-linked promoter region (*5HTTLPR*), Gamma-aminobutyric acid type A receptor subunit beta3 (*GABRB3*).

## Data Availability

Not applicable.
